# Habitat Use by Fishes in Coral Reefs, Seagrass Beds and Mangrove Habitats in the Philippines

**DOI:** 10.1371/journal.pone.0065735

**Published:** 2013-08-20

**Authors:** Kentaro Honda, Yohei Nakamura, Masahiro Nakaoka, Wilfredo H. Uy, Miguel D. Fortes

**Affiliations:** 1 Akkeshi Marine Station, Field Science Center for Northern Biosphere, Hokkaido University, Aikappu, Akkeshi, Hokkaido, Japan; 2 Graduate School of Kuroshio Science, Kochi University, Nankoku, Kochi, Japan; 3 Institute of Fisheries Research and Development, Mindanao State University at Naawan, Misamis Oriental, The Philippines; 4 The Marine Science Institute, CS, University of the Philippines-Diliman; Diliman, Quezon City, The Philippines; Smithsonian's National Zoological Park, United States of America

## Abstract

Understanding the interconnectivity of organisms among different habitats is a key requirement for generating effective management plans in coastal ecosystems, particularly when determining component habitat structures in marine protected areas. To elucidate the patterns of habitat use by fishes among coral, seagrass, and mangrove habitats, and between natural and transplanted mangroves, visual censuses were conducted semiannually at two sites in the Philippines during September and March 2010–2012. In total, 265 species and 15,930 individuals were recorded. Species richness and abundance of fishes were significantly higher in coral reefs (234 species, 12,306 individuals) than in seagrass (38 species, 1,198 individuals) and mangrove (47 species, 2,426 individuals) habitats. Similarity tests revealed a highly significant difference among the three habitats. Fishes exhibited two different strategies for habitat use, inhabiting either a single (85.6% of recorded species) or several habitats (14.4%). Some fish that utilized multiple habitats, such as *Lutjanus monostigma* and *Parupeneus barberinus*, showed possible ontogenetic habitat shifts from mangroves and/or seagrass habitats to coral reefs. Moreover, over 20% of commercial fish species used multiple habitats, highlighting the importance of including different habitat types within marine protected areas to achieve efficient and effective resource management. Neither species richness nor abundance of fishes significantly differed between natural and transplanted mangroves. In addition, 14 fish species were recorded in a 20-year-old transplanted mangrove area, and over 90% of these species used multiple habitats, further demonstrating the key role of transplanted mangroves as a reef fish habitat in this region.

## Introduction

In the tropics, seagrass beds and mangroves are formed in the shallow reef flat zone and the near coastline/estuarine region, respectively. Habitat-specific fish species inhabit either of these habitats, whereas some coral reef fishes, such as Lutjanidae, Haemulidae, Lethrinidae, Scaridae, Siganidae, and several other families, utilize these habitats as their nursery grounds [1]–[13]. In addition, several fish species show diel movements among these habitats for feeding or shelter [14], [15]. Local connectivity by fishes and its importance among coral, seagrass, and mangrove ecosystems have received a great deal of attention in recent years [16]–[19]. Previous studies have indicated that the intensity and characteristics of connectivity by reef fishes widely fluctuate depending on regional differences and/or geographical conditions (see [18]), while the intensity also weakens depending on the distance between habitats [20]–[22]. Although several studies have been conducted in various regions under different conditions, few have been performed in Southeast Asian countries relative to other regions such as the Caribbean and Australia (see [18]). Furthermore, only a few studies have evaluated differences in the intensity or effectiveness of connectivity by reef fishes between non-estuarine and transplanted mangroves in the Indo-Pacific [11], [21]–[24].

The rates of environmental deterioration at various scales and unabated overfishing continue to increase worldwide, resulting in reductions of fishery resources. Open access to fishing grounds [25] has become a popular trend, leaving resources at the brink of collapse [26], [27]. While fish resources face threats of the loss of both biodiversity and stock replenishment, the degradation of coral reef ecosystems has also become a huge social problem (e.g., [28]–[30]). The establishment of marine protected areas (MPAs) is seen as an effective tool to protect coastal habitats and to enhance nearshore fisheries, especially in tropical regions (e.g., [31]–[33]). When establishing a MPA to protect fishery resources, the life history and habitat use of target species must first to be clearly determined. If target fishes exhibit ontogenetic habitat shifts (i.e., habitat changes with growth stages) or if fishes move daily among different habitats for feeding or shelter, all habitats being used may be equally important, regardless of scale and type; therefore, each of these habitats must be included in the establishment of MPA boundaries. Even when establishing a MPA for biodiversity conservation, the inclusion of multiple habitats within a MPA could further enhance its effectiveness, as such a MPA may be able to protect not only habitat-specific species, but also those that inhabit multiple habitats. For these reasons, understanding the interconnectivity of reef fishes among different habitats is a key requirement for making effective management plans in coastal ecosystems, particularly for determining the component habitat structures in a MPA.

Most coastal areas in the Philippines are located in the Coral Triangle, an area known for the highest biodiversity of coral worldwide [34], [35]. However, habitat loss along the Philippine coasts has remarkably increased in recent years [36]–[38]. In particular, more than half of natural mangroves had disappeared by 1994, mainly due to the establishment of fish ponds [36], [39]. Furthermore, seagrass beds are drastically decreasing, even though these habitats serve as equally important fishing grounds as coral reefs for various commercially important species such as Lethrinidae or Siganidae [40]–[42]. Since the 1930s–1950s, mangrove replantation projects have been implemented and have emphasized the participation of local communities in the Visayas region [43]. In recent years, however, sustaining the health of fishery resources has become difficult in the Philippines, particularly due to poor environmental governance and lack of effective, coherent monitoring programs. This situation eventually led to increased overfishing and other forms of environmental deterioration, which consequently became the topmost increasing concern regarding the proper management of fishery resources and MPAs [44]–[46]. From 1967 to the present, nearly 1,000 MPAs have been established in the Philippines [46]. Upon careful review, most of them focus on coral reefs, and those that incorporate multiple habitats number relatively few. In addition, the implications of connectivity among different habitats have been underexplored and are poorly understood [47], [48]. In the Philippines, several studies have evaluated the effectiveness of MPA management in terms of fishery regulations (e.g., [49], [50]). Furthermore, the effectiveness of fishery resource conservation was verified by a series of studies in the Sumilon and Apo islands (e.g., [51]–[53]). These studies documented the effects of MPAs on the enhancement of fisheries components; however, they only focused on one type of ecosystem (i.e., coral reefs) and disregarded important effects of the multiple habitats used by some commercially important fish species. If the present study can determine the specific features of each habitat, their connectivity, and corresponding importance, then our findings would be valuable for fishery resource conservation and management in regions of the Philippines. Furthermore, we also compared the transplanted mangroves to other types of habitats; such comparisons have been rare in previous studies.

The present study was designed to address differences in the pattern of habitat use by fishes, with a focus on commercial fishery species, among coral, seagrass, and mangrove habitats in the Philippines and whether transplanted and natural mangroves are used as common habitats for adult fishes and/or as potential nursery habitats for juveniles [54]. Based on our results, we further discuss the importance of including multiple habitats within MPAs.

## Materials and Methods

### Study Design

**Figure 1:**
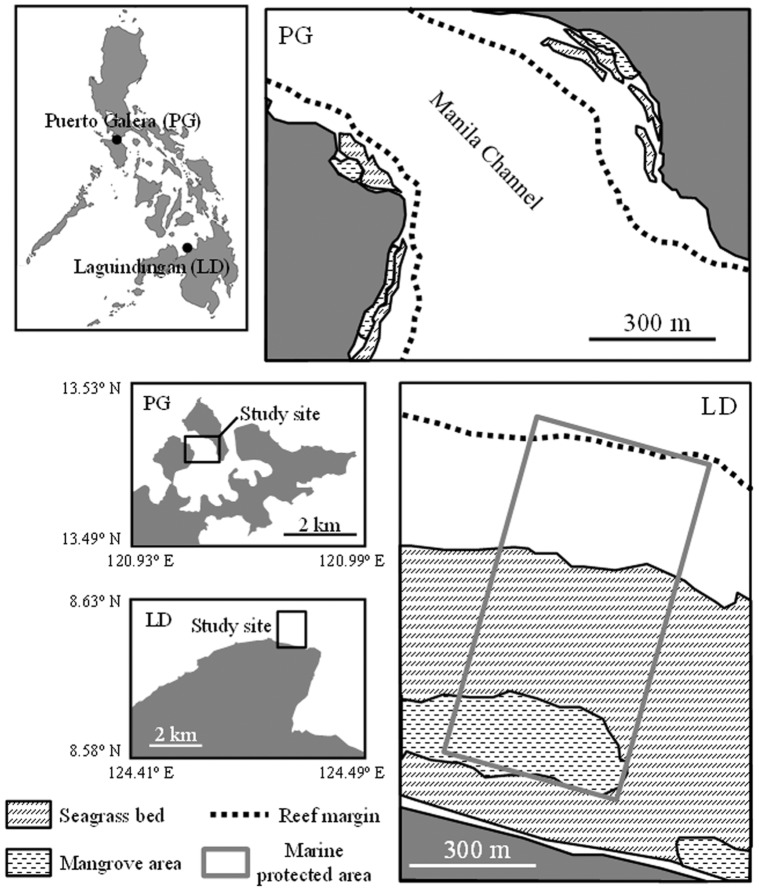
The two study sites in the Philippines.

Field surveys were conducted semiannually for 2 years (2010 and 2011) during months representing the rainy season (September) and the dry season (March) at Puerto Galera (PG; 13°30′ N, 120°57′ E) off of northern Mindoro Island, and at Laguindingan (LD; 8°37′ N, 124°28′ E) off of northern Mindanao Island, the Philippines (Figure 1). The study site at PG was situated in a fringing reef with the reef flat zone located along both the western and eastern side of Manila Channel within Puerto Galera Bay. The study site at LD was located in a fringing reef where the reef flat zone faces the open sea. The MPAs of PG (entire study site) and LD were established in 2006 and 2002, respectively, both with a strict no-take-zone policy (Figure 1). Coral reefs at both sites are composed of hermatypic corals (e.g., tabular and branching *Acropora*; living coral coverage, >80%), which are more abundant near the reef margins. Dominant seagrass species at PG were *Thalassia hemprichii* (15.9% of cover), *Halodule pinifolia* (15.0%), and *Cymodocea rotundata* (12.0%), whereas *T. hemprichii* (63.6%) and *Enhalus acoroides* (4.3%) were dominant at LD. The mean (± SD) canopy heights at PG and LD were 6.4±5.0 cm (*n* = 148 quadrats) and 11.4±3.4 cm (*n* = 156 quadrats), respectively. *Rhizophora apiculata* and *Sonneratia* sp. were the dominant mangrove species at PG, whereas only *R. apiculata* was present at LD. Mangrove areas at both sites were composed of clear-water non-estuarine mangrove (Figure 1). The mangroves at LD have been planted along seagrass beds near the shoreline since 1992, and they presently form a band of young and mature trees that protect the coastal communities from strong winds (Honda, personal communication). Fish distribution patterns among coral reefs, seagrass beds and mangrove areas were assessed during each season using an underwater visual transect survey method. In each habitat, seven 1×20-m (20 m^2^) belt transects were established haphazardly using a scaled rope (see also [8], [9]). Transects were separated from one another by at least 5 m, In PG, three and four line transects were established in each habitat along the western and eastern side, respectively, of Manila Channel. All fish visual censuses (FVCs) were conducted in daytime between 08∶00 and 16∶00 h, and fishes were identified to the lowest taxonomic level whenever possible. Individual fish size (total length) was also recorded underwater using a ruler attached to the recording slate. In coral reefs, FVC were conducted via SCUBA or snorkeling depending on the water depth (2.0–8.0 m). In seagrass beds (0.5–1.0 m deep at low tide, 1.5–2.0 m at high tide) and mangrove areas (0.5–1.0 m at low tide, 1.0–1.5 m at high tide), only snorkeling was used and FVCs were conducted when the depth ranged from 1.0 to 1.5 m to avoid tidal effects [55]. Visibility within the water at any transect generally exceeded 7 m. Sea surface water temperature at PG and LD was 30.0°C and 30.4°C in September and 27.8°C and 28.8°C in March, respectively. Salinity at both sites was about 34‰, and no estuaries were present near either site. All methods utilized in the present study were conducted under the permit requirements of the municipal government of PG and the Barangay Tubajon in LD.

### Data Analysis

At both sites, data collected from each habitat type were analyzed for species composition and density. Because assumptions of homogeneity of variance could not be met by some data even after transformations, nonparametric Steel–Dwass tests were used to determine whether species and individual numbers of fishes differed among habitats for each site, month, and year (see also [9]). Moreover, these variables were compared between PG and LD mangrove areas in each sampling month using Mann–Whitney *U*-tests. Family composition of species and individuals in each habitat at each site was also estimated.

The similarity of fish assemblages among habitats was examined using data from the seven transects within each habitat for every month. The Chao index [56] was used for this analysis and results were visualized using nonmetric multidimensional scaling (NMDS). Similarity tests among four variables (year, month, site and habitat) were conducted using nonparametric multivariate analysis of variance (NPMANOVA; *α = *0.05). All statistical analyses were performed using the “vegan” package of R ver. 2.14.1 (R Development Core Team).

Counts of each fish species that occurred in two or more habitats were analyzed to verify the presence of fish that use multiple habitats. To avoid incidental detection, instances of only one individual- of a species recorded in a habitat, along with unidentified species, were excluded from analysis. Moreover, based on the commercial fishery species listed in Fishbase [57], the number of commercial fish species utilizing a particular habitat type or a combination of habitats was determined. Using these data, the habitat or combination of habitas favored by a large number of commercial species was evaluated. Here, commercial species included species listed as “highly commercial” or “commercial” in Fishbase, while other categories, such as “minor commercial,” “subsistence fisheries,” “of no interest,” and “no information,” were not regarded as commercial species. Here, *Pomacentrus lepidogenys*, which was categorized as “highly commercial” in Fishbase, was considered a noncommercial species together with other pomacentrids, because it is highly unlikely that this species was of high fishery importance in the Philippines. Moreover, for cases in which fish exhibited possible ontogenetic habitat shifts, the size distribution pattern in each habitat was visualized. Fish species were considered to undergo possible ontogenetic habitat shifts based on individual counts or mean length. If the individual count of a fish species reached 10 or more within juvenile habitats (seagrass and/or mangrove) and five or more in coral reefs, then this fish species was considered to undergo a potential ontogenetic habitat shift. In addition, if the mean total length of a fish species from the coral reef was significantly longer than that in the juvenile habitat (Mann–Whitney *U*-test, *α = *0.05), then such a fish species may also exhibit an ontogenetic habitat shift. Species belonging to Atherinidae and Gobidae families were excluded from all analyses because they are pelagic and small cryptic fishes, respectively.

## Results

### Fish Assemblage Structure

**Figure 2:**
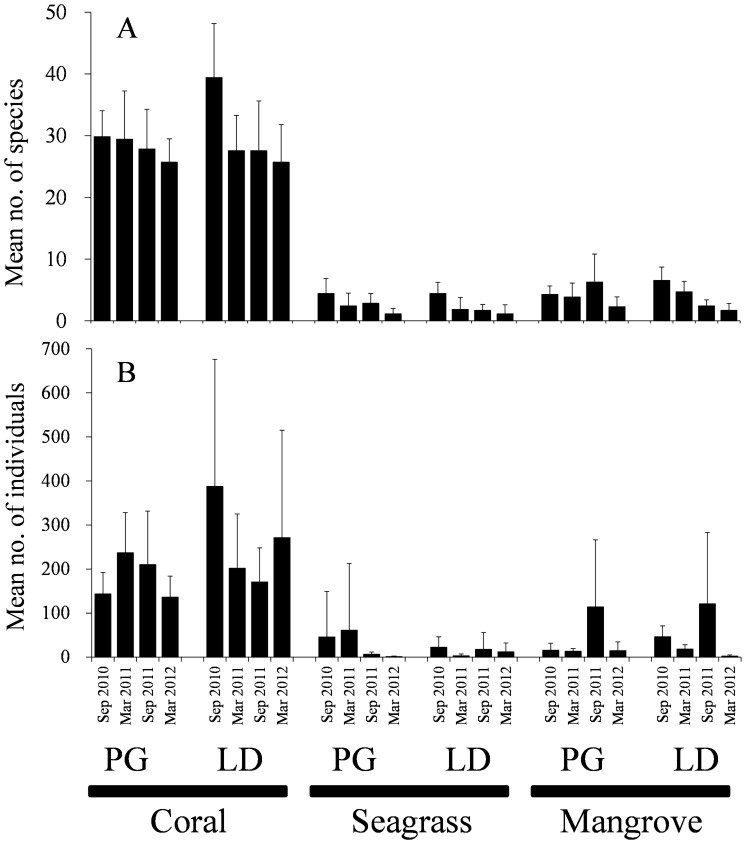
Mean number of fish species (A) and individuals (B) per transect (1×20 m) during sampling months in each habitat at each study site. Error bars are standard deviations of *n = *7 transects. PG and LD represent Puerto Galera and Laguindingan, respectively.

In total, 15,930 individuals, belonging to 265 species in 45 families were recorded (Table S1). In coral reefs, 12,305 individuals comprising 234 species in 37 families were recorded. In contrast, fewer fish were recorded in seagrass beds (1,198 individuals belonging to 38 species in 18 families) and mangrove areas (2,426 individuals belonging to 47 species in 24 families). The mean numbers of species and individuals per transect in coral areas at both PG and LD sites were significantly higher than those in seagrass and mangrove habitats (*P*<0.05), with four exceptions for the number of individuals (seagrass beds in September 2010 and March 2011 at PG and mangrove areas in September 2011 in both PG and LD; Figure 2). Seagrass and mangrove habitats did not significantly differ in terms of either the number of species or individuals (*P*>0.05), although the numbers of both species and individuals at LD in March 2011 differed significantly between seagrass and mangrove habitats. Neither the number of species nor individuals (*P*>0.05) significantly differed between PG and LD mangroves, except during September 2011, when the number of species in mangroves was higher at PG than that at LD.

**Figure 3:**
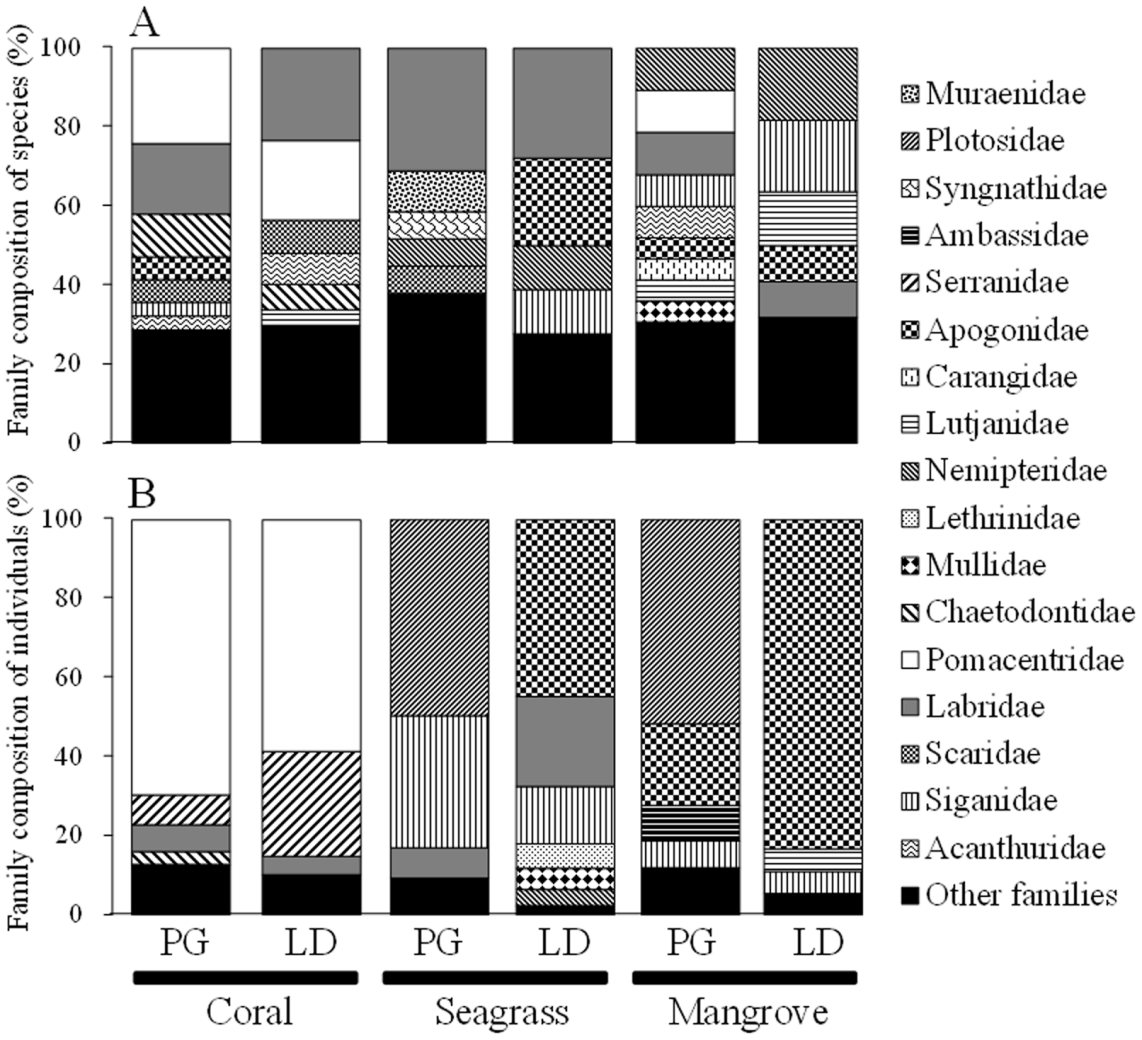
Relative family composition of fish species (A) and number of individuals (B) in the different habitats at each study site.

The three most dominant families in terms of the number of species in PG coral reefs were Pomacentridae, Labridae, and Chaetodontidae (Figure 3a). Dominant families in LD were Labridae and Pomacentridae, and the combined abundance of these families accounted for more than 40% at both PG and LD. For fish in seagrass beds at PG, Labridae accounted for about 30% of all species, followed by Muraenidae, Syngnathidae, Nemipteridae, and Scaridae, in that order. Labridae and Apogonidae together accounted for more than half of the fish species in LD seagrass beds. In mangrove areas, the three most dominant families were Nemipteridae, Pomacentridae and Labridae at PG and Nemipteridae, Siganidae and Lutjanidae at LD. Pomacentridae (represented by *Chromis ternatensis*, *Acanthochromis polyacanthus*, and *Pomacentrus moluccensis*) was the most dominant family in terms of the number of individuals in coral reefs at both PG (69.5%) and LD (58.6%), followed by Seranidae (represented by *Pseudanthias huchti*; Figure 3b). Fish family composition in seagrass beds differed between PG and LD. Only two species, *Plotosus lineatus* (Plotosidae) and *Siganus spinus* (Siganidae), accounted for over 80% of all fish species at PG. In contrast, at LD, Apogonidae (represented by *Apogon ceramensis*), Labridae (represented by *Halichoeres argus* and *Halichoeres scapularis*), and Siganidae (represented by *S. spinus*) were the three most dominant families, together comprising 80% of fish individuals. In mangrove areas, Plotosidae (represented only by *P.lineatus*) and Apogonidae (represented by *Sphaeramia orbicularis* and *A. ceramensis*) together accounted for about 70% of fish individuals at PG. Apogonidae also represented by *S. orbicularis* and *A. ceramensis*) accounted for more than 80% of fish individuals at LD.

At the species level, 199 species were unique to coral reefs, whereas nine and 15 species were unique to seagrass beds and mangrove areas, respectively (Table S1). At the family level, 14 families were only recorded in coral reefs: Holocentridae, Aulostomidae, Centriscidae, Pseudochromidae, Priacanthidae, Caesionidae, Chaetodontidae, Kyphosidae, Blennidae, Gobiesocidae, Zanclidae, Balistidae, Ostraciidae, and Diodontidae. Five families were only recorded in the mangrove areas: Mugilidae, Hemiramphidae, Belonidae, Ambassidae, and Terpontidae. No families were unique to seagrass habitats.

**Figure 4:**
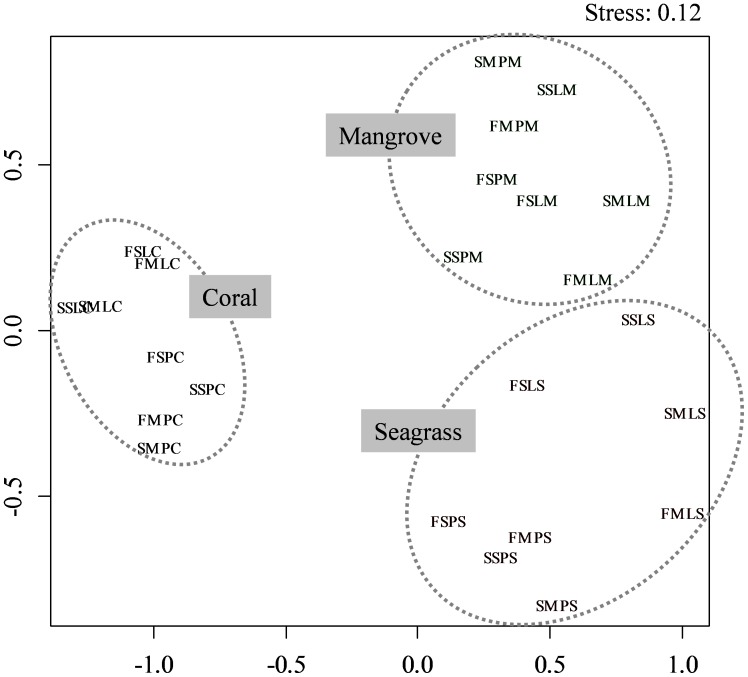
Nonmetric multidimensional scaling (NMDS) of data from all seven transects in each habitat at each site in each month of each year. Each data set is represented by four letters in the order from Year (First or Second), Month (September or March), Site (Puerto Galera or Laguindingan), and Habitat (Coral reef, Seagrass bed, or Mangrove area).

Similarity indices revealed that fish communities could be divided into three large groups (coral, seagrass, and mangrove habitat types) regardless of sampling month and site (Figure 4). Results of similarity tests using NPMANOVA revealed a highly significant difference among habitats (*F* = 11.28, *P*<0.001). Other variables, such as sampling period (year, month), did not significantly affect patterns of fish structure (*F* <2.0, *P*>0.05), although a marginal difference was observed between sites (*F* = 1.92, *P* = 0.08).

### Fishes Utilized Multiple Habitats

In total, 29 fish species accounting for 14.4% of species abundance were recorded in multiple habitats (Table 1). Six species were recorded in both coral and seagrass habitats and three of these belonged to Labridae (Table S1). Nine species were recorded in both coral and mangrove habitats: three species belonged to Pomacentridae, while Lutjanidae and Siganidae were represented by two species each. Six species were observed in both seagrass and mangrove habitats, and each belonged to different families. Eight species were recorded in all three habitats, and Labridae and Siganidae were represented by two species each.

**Table 1:** Number of fish species at all sampled sites for each habitat and each combination of different habitats.

Habitat number	Observed habitat	All species			Commercial species		
		Puerto Galera	Laguindingan	Both sites	Puerto Galera	Laguindingan	Both sites
1	Coral reef only	110 (74.3)	103 (83.1)	157 (78.1)	17 (54.8)	20 (71.4)	27 (62.8)
2	Seagrass bed only	5 (3.4)	4 (3.2)	6 (3.0)	2 (6.5)	0 (0.0)	2 (4.7)
3	Mangrove area only	14 (9.5)	1 (0.8)	9 (4.5)	6 (19.4)	0 (0.0)	4 (9.3)
4	Both coral reef & seagrass bed	5 (3.4)	3 (2.4)	6 (3.0)	0 (0.0)	2 (7.1)	1 (2.3)
5	Both coral reef & mangrove area	7 (4.7)	5 (4.0)	9 (4.5)	3 (9.7)	3 (10.7)	3 (7.0)
6	Both seagrass bed & mangrove area	3 (2.0)	7 (5.6)	6 (3.0)	2 (6.5)	2 (7.1)	2 (4.7)
7	All three habitats	4 (2.7)	1 (0.8)	8 (4.0)	1 (3.2)	1 (3.6)	4 (9.3)
4–7	Multiple habitats	19 (12.8)	16 (12.9)	29 (14.4)	6 (19.4)	8 (28.6)	10 (23.3)
3, 5–7	Mangrove area	28 (18.9)	14 (11.3)	32 (15.9)	12 (38.7)	6 (21.4)	13 (30.2)
2–7	All except coral reef only	38 (25.7)	21 (16.9)	44 (21.9)	14 (45.2)	8 (28.6)	16 (37.2)
1–7	All	148 (100)	124 (100)	201 (100)	31 (100)	28 (100)	43 (100)

Percentages of totals are shown in parentheses. Instances for which only one individual was recorded in a habitat for each species were excluded (i.e., numbers differ from those in Table S1). All unidentified species were also excluded.

For commercial species, the total number of species in coral reefs was 27 (62.8%), which was relatively greater than the numbers found in seagrass beds (two species, 4.7%) and mangrove areas (four species, 9.3%; Table 1). Ten commercial species (23.3%) were recorded in multiple habitats. Sixteen commercial species used multiple habitats or exclusively used either seagrass beds or mangrove areas, accounted for 37.2% of commercial species (i.e., “all except coral reef only” group in Table 1).

Fourteen fish species were recorded in the transplanted mangrove area at LD, 13 of which also utilized coral and/or seagrass habitats (Table 1). Even though minimal differences were found between PG and LD in terms of the numbers of total species, commercial species, and multiple habitat users, the number of all fish species that were mangrove users, including those with commercial value, was twice higher at PG than at LD (Table 1). In addition, PG and LD differed greatly in the number of species observed in only mangrove habitat (Table 1): *S. orbicularis* was the only species recorded as a mangrove only user in LD, whereas 14 species were exclusively recorded in the mangrove areas at PG.

**Figure 5:**
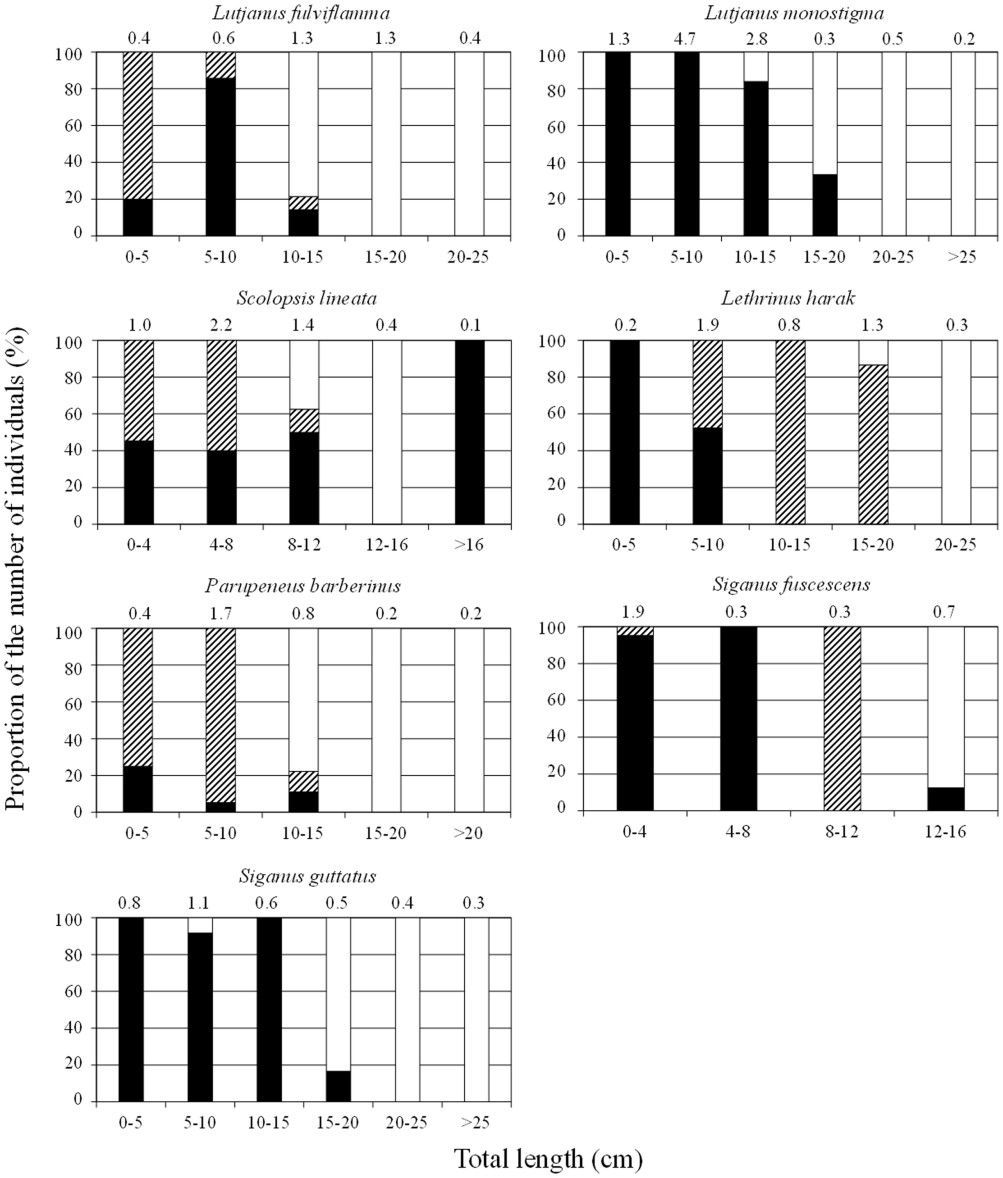
Relative abundance of the seven fish species in coral reefs (white), seagrass beds (hatched), and mangrove areas (black) across habitats according to class sizes using pooled data from Puerto Galera and Laguindingan. See Table S1 for size distribution of fishes in each habitat for every site. Mean individual numbers per 1,000 m^2^ during the period are given above each size-class column.

In the present study, seven species (Lutjanus fulviflamma, Lutjanus monostigma, Scolopsis lineata, Lethrinus harak, Parupeneus barberinus, Siganus fuscescens, and Siganus guttatus) exhibited possible ontogenetic habitat shifts from seagrass beds and/or mangrove areas to coral reefs (Figure 5). Of these seven species, multiple adult-sized individuals of only L. harak (n = 8) occurred in seagrass beds.

## Discussion

The present study revealed that fish assemblage structure varied significantly among coral, seagrass, and mangrove habitats at the study sites, although differences between seagrass and mangrove habitats in terms of species richness and abundance were not significant (Figures 2, 4). The 199 fish species recorded only in coral reefs was accounted for approximately 75% of all fish species recorded, whereas only nine and 15 fish species exclusively utilized in seagrass and mangrove habitats, respectively (Table S1). Although the majority of fish species was found in coral reefs, the other habitats also yielded several unique species. The fact that five families of fish were found only in mangrove habitats emphasizes the need to conserve multiple habitats even without considering connectivity. Different habitats exhibit different environmental conditions and fish assemblage structures; thus, managing all of these habitats can serve as a very effective method of conserving coastal biodiversity.

Even though each habitat exhibited different fish compositions, many fish species were found to use multiple habitats, and these fish could be categorized into two groups. The first group includes those species that generally did not change habitat preference after settlement although they did inhabit more than one habitat (also see [19]). The second group includes fishes that use seagrass and/or mangrove habitats as their feeding or shelter grounds at the adult stage or as nursery grounds in the juvenile stage. Adult-sized *Lutjanus griseus* and mullids are examples of fish that migrate daily into seagrass/mangrove habitats for feeding, similar to the observations of Nakamura and Tsuchiya [8] and Luo et al. [15]. In this study, several adult-sized *L. harak* were observed not only in coral reefs but also in seagrass beds. Although we could not determine if these individuals migrated between habitats or if each habitat harbored its own population, adult individuals of coral fishes have often been observed in seagrass beds and/or mangrove areas in previous studies (e.g., [11], [24]). Adult *P. barberinus* and *S. guttatus* were sometimes observed in seagrass beds at the study sites, although these fish were not recorded in the transect survey. Feeding behavior of *L. harak* and *P. barberinus* was also observed in seagrass beds (Honda, personal observation). Moreover, seven species were confirmed to use seagrass and/or mangrove habitats during the juvenile stage. Even though fish species commonly exhibit ontogenetic habitat shifts (see [18], [19]), it is also important to recognize that the juvenile fish of some species were found only in seagrass beds and/or mangroves (e.g., [2], [9]). Such habitat fidelity is considered to reduce the likelihood of fish flexibility or opportunism in habitat use.

More than 37% of the commercial fish recorded in this study utilized seagrass and/or mangrove habitats or one of these habitats in combination with coral reefs, and more than 34% of the fish that utilized multiple habitats in this study were commercial species (Table 1). In addition, six of seven species that exhibited possible ontogenetic habitat shifts (the exception being *S. lineata*) were commercial species. Based on previous reports, many of the species that exhibit ontogenetic habitat shifts, as well as those species that migrate among different habitats in their adult stage, have fishery value (e.g., [2], [12], [15]). Thus, the inclusion of adjacent seagrass beds and mangrove areas connected to coral reefs in the same MPA offers several important benefits: increased carbon dioxide fixation or sequestration by seagrasses and mangroves [58], buffering against disasters such as high waves [59], enhanced conservation of biodiversity of organisms, and increased sustainability of fishery resources [21], [22], [42], [60].

Regrettably, seagrass beds and mangrove forests are disappearing worldwide [61]–[63]; consequently, the species richness and biomass of fishes and invertebrates decrease with such habitat losses [16], [64]. In the present study, 14 fish species that utilize mangroves were recorded in the transplanted mangrove area, and most of these species were multiple habitat users (Table 1), indicating that both natural and transplanted mangroves play an important role as habitat for some reef fishes. Moreover, this finding suggests that transplanting mangroves can be useful in terms of fishery resource conservation and recovery. The total number of fish species considered as mangrove users at LD was half in the value observed at PG, even though the two sites did not greatly differ in the number of multiple habitat users (Table 1). Almost two decades have passed since mangroves were transplanted at LD; however, much more time may be needed for the colonization of fish species that are dependent on mangroves for their recovery and population replenishment, as only a few natural mangroves exist nearby as sources of populations of fish species. The fact that *S. orbicularis* was the only fish species unique to mangrove areas at LD supports this hypothesis.

Blaber and Milton [65] and Thollot [66] reported that fish species diversity in clear-water mangroves was lower than that in estuarine mangroves. In most cases, estuarine mangroves are surrounded by more simply structured habitats, such as mud or sand habitats, whereas clear-water non-estuarine mangroves connected to coral reefs exhibit a more complex structure. Such differences in the complexity of the surrounding habitats may affect the structure of species diversity between clear-water non-estuarine mangroves and estuarine mangroves [24]. In addition, a few reports have indicated that clear-water non-estuarine mangroves in the Indo-Pacific serve as juvenile habitat for reef fishes [11]. Barnes et al. [24] compared fish assemblage structure between coral reefs and clear-water non-estuarine mangrove areas near Orpheus Island in the Great Barrier Reef, and they found that no specific reef fish species used clear-water non-estuarine mangroves as their juvenile habitats. Nevertheless, we found that seven reef fish species exhibited possible ontogenetic habitat shifts from clear-water non-estuarine mangroves to coral reefs. This finding strongly indicates that clear-water non-estuarine mangroves in the Indo-Pacific function as the juvenile habitat of some reef fishes, similar to mangroves in the Caribbean region (e.g., [1], [67]) and estuarine mangroves in the Indo-Pacific [6], [9], [68].

Human population near coastal areas in the tropics are expected to double in the next 50–100 years, ultimately leading to increased fishing pressure and therefore accelerated biodiversity loss and depletion of fishery resources [69]. Even though several limited MPAs contribute to fishery resource conservation and recovery (e.g., [51], [70]), coastal fisheries are not effectively managed in most coastal areas in Southeast Asian regions, including the Philippines [46]. This situation will presumably get worse in the future with increases in the human population. In the tropics, not only overfishing but also juvenile habitat loss, followed by decreases in the survival rate of juveniles, have strongly contributed to declines in fishery resources in recent years. The conservation and development of juvenile habitats would delay the exhaustion of such resources.

## Supporting Information

Table S1 **Total number of individuals and size range of 265 species in three habitats at two study sites during the study period.** PG, LD, and TL indicate Puerto Galera, Laguindingan, and total length, respectively.
(XLSX)
